# Supplementary material for: Exogenous Sodium and Calcium Alleviate Drought Stress by Promoting the Succulence of Suaeda salsa
Source: Plants (Basel). 2024 Mar 4;13(5):721. doi: 10.3390/plants13050721 (PMC10934976; doi:10.3390/plants13050721)
Supplement: Supplementary file 1 [file plants-13-00721-s001.zip › plants-2790061-supplementary.pdf]

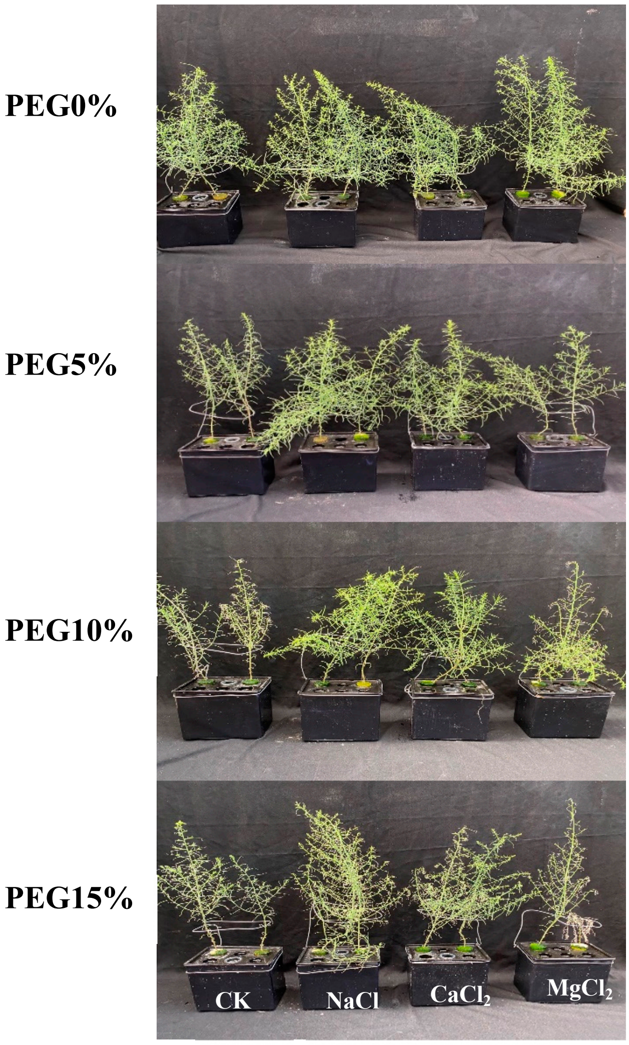

Figure S1: Effects of Polyethylene Glycol (PEG) Concentration and Different Salt Ions on the growth of *S. salsa*.
